# Supplementary material for: Mechanically induced integrin ligation mediates intracellular calcium signaling with single pulsating cavitation bubbles
Source: Theranostics. 2021 Apr 7;11(12):6090–104. doi: 10.7150/thno.56813 (PMC8058710; doi:10.7150/thno.56813)
Supplement: Supplementary file 1 — Supplementary figures and tables. [file thnov11p6090s1.pdf]

Supporting Information

Mechanically induced integrin ligation mediates intracellular calcium signaling with single pulsating cavitation bubbles

Fenfang Li, Tae Hyun Park, George Sankin, Christopher Gilchrist, Defei Liao, Chon U Chan, Zheng Mao, Brenton D. Hoffman*, Pei Zhong**

Supplementary Information Text

Single cavitation bubble (SCB)-generated shear flow: shear stress estimation at different γ. Based on previous study with similar bubble dynamics, the shear stress τ near the substrate at fixed γ can be estimated from the velocity field [1]:

$$\tau\,(\mathrm{t}) = \rho\sqrt{\frac{\nu}{\pi}}\int_0^t \frac{\partial V(t\prime)}{\partial t\prime}\frac{dt\prime}{\sqrt{t-t\prime}}\ ,$$

which can be written in a discrete from:

$$\tau\,(\mathrm{t}_m) = \rho\sqrt{\frac{\nu}{\pi}}\left(\sum_{n=1}^{m}\frac{V_n-V_{n-1}}{\Delta t}\frac{\Delta t}{\sqrt{\mathrm{t}_m-t_{n-1}}}\right) = \rho\sqrt{\frac{\nu}{\pi}}\left(\sum_{n=1}^{m}\frac{V_n-V_{n-1}}{\sqrt{\mathrm{t}_m-t_{n-1}}}\right) \qquad (1)$$

where t_n and V_n is the time and velocity for the recorded image frame # n, bubble is generated at frame n=0, Δt=0.5 μs, ρ is the density of the liquid medium (1000 kg m^{-3}), ν is the liquid kinematic viscosity (0.896 x 10^{-6} m^2 s^{-1})

Moreover, considering the impulsive nature of the shear flow induced by single bubbles, we calculated shear stress integral (SSI) that incorporates the contribution of both the amplitude and the duration of the shear stress by:

$$SSI = \int_{t1}^{t2}\tau^{\beta}dt, \qquad (2)$$

Where t is the time, and t_1 and t_2 delineate the lower and upper integration limits during the shear flow. Previous studies have indicated that strain or stress integral may be appropriate for gauging the membrane poration under dynamic shear stress with a value of β ~ 2 at the single cell level [2-4]. It is worth noting that although shear stress impulse is reported in previous study [5, 6], intercellular calcium wave is studied there in confluent cell monolayer, which will propagate from cell to cell with time.

The estimated shear stress by using PIV data and Eq.1 is shown in Figure S2. Within γ=1.5-1.8, we found similar shear stress impulse, and particularly similar shear stress integral for two bubbles that represent the upper and lower limit of the bubble size used in this study.

Data pooling over the range of γ = 1.5-1.8. Figure S7 depicts the dependency of cell Ca^{2+} response $(F_p-F_0)/F_0$ on the normalized standoff distance γ for the mechanistic studies shown in Fig. 6A&B of the main text. Overall, no strong dependence is observed within the non-injury range of γ = 1.5-1.8.

Spatial evolution of intracellular Ca^{2+} signaling in P1KO cells when beads are not used. When there was membrane poration (Fig. 2A), Ca^{2+} wave initiated then propagated from the poration site, similar to our previous study [7]. When Ca^{2+} response was evoked without membrane poration (Fig. 2B), the time evolution of Ca^{2+} response in edge, middle and center ROI (Fig. S8) showed similar regional differences as the cell treated with RGD beads (Fig. 4B). It indicates Ca^{2+} response initiated from the cell edge, where

the focal adhesion and integrin were located. This result supports our hypothesis that integrin-ligation on substrate ECM plays an important role in eliciting non-injury Ca^{2+} response in P1KO cells.

It is worth noting that without beads, there is small chance to get non-injury Ca^{2+} response. RGD beads enhance the local drag on P1KO cells and transmit it into cells through the RGD-integrin link, thus more efficiently evoking integrin ligation to substrate ECM.

General observation of cell spread area alteration with TB induced Ca^{2+} response. Transient increase, reduction and recovery of cell spread area are also observed in TB treated HeLa cells that show non-injury Ca^{2+} response from our previous study [7], see Figure S4 (A-B). The results again suggest that Ca^{2+} response is initiated during the transient increase of cell spread area following TB treatment (see the enlarged view). The intracellular Ca^{2+} elevation is followed by cell spread area reduction (see the green dashed lines that reach the peak value successively), and the intracellular Ca^{2+} decay preceded the cell spread area recovery. These results suggest Ca^{2+} response is driving reduction and recovery of the cell spread area.

We found a similar trend of Ca^{2+} response driving the reduction and recovery of the cell spread area in TB elicited injury Ca^{2+} response w/o beads, see Figure S4C. Interestingly, for these injury Ca^{2+} response, there is no obvious increase of the cell spreading area following the TB treatment, consistent with our observations for SCB treated HEK293T cells with injury (Figure 5F).

A summary of the maximum cell spread area reduction in relation to the amplitude of the Ca^{2+} response is shown in Figure S4D. With increased amplitude of Ca^{2+} signaling, the individual cell's peak spread area reduction gradually transits from no Ca^{2+} response (black symbols) to non-injury Ca^{2+} response (blue symbols), and finally injury Ca^{2+} response (red symbols). The data also reveals that the peak spread area reduction is below 5% for all the no Ca^{2+} response cases, within 5-12% for the majority of the non-injury Ca^{2+} response cases, and above 12% for most of the injury Ca^{2+} response cases, similar to the results for SCB treated HEK293T P1KO cells shown in Figure 5G.

Error analysis for calcium response amplitude and cell spread area reduction. The error in the normalized calcium response amplitude $\boldsymbol{f}$ and cell spread area reduction $\boldsymbol{A}$ is calculated by error propagation of

$$\boldsymbol{f} = \frac{F_p - F_0}{F_0} = \frac{F_p}{F_0} - \mathbf{1}\ , \quad \boldsymbol{A} = \frac{S_0 - S_p}{S_0} = \mathbf{1} - \frac{S_p}{S_0}$$

The uncertainty, where the related variables are baseline and peak value of fura2 ratio $\boldsymbol{F_0}$, $\boldsymbol{F_p}$, and spread area of the cell $\boldsymbol{S_0}$, $\boldsymbol{S_p}$. Let $\boldsymbol{u}$ denotes the uncertainty of the respective variables, the uncertainty of $\boldsymbol{f}$ can be derived as:

$$u_f = \sqrt{(\frac{\partial f}{\partial F_p})^2 {u_{F_p}}^2 + (\frac{\partial f}{\partial F_0})^2 {u_{F_0}}^2} = \sqrt{(\frac{1}{F_0})^2 \ {u_{F_p}}^2 + (\frac{-F_p}{{F_0}^2})^2 {u_{F_0}}^2} = \frac{1}{F_0}\sqrt{{u_{F_p}}^2 + (\frac{F_p}{F_0})^2 {u_{F_0}}^2}$$

As $u_{F_p} \sim u_{F_0}$ & $\frac{F_p}{F_0} = f + 1$

$$u_f = \frac{u_{F_0}}{F_0}\sqrt{1 + (f + 1)^2} \qquad (3)$$

Similarly, the uncertainty of $\boldsymbol{A}$:

$$u_A = \frac{u_{S_0}}{S_0}\sqrt{1 + (1 - A)^2} \qquad (4)$$

F_0 and S_0 are the averaged value of the baseline, their SD and SEM can be easily calculated. Therefore, using eq.[3] and eq.[4] we can find the SEM of the normalized calcium response amplitude f and cell spread area reduction A. The estimated error are shown in Figure 5, Figure S4D and Figure S6.

Summary on the activation of piezo1 from previous studies using ultrasound or shear flow. A list of studies reported activation of Piezo1 by ultrasound or shear flow, where the stimulation duration is mainly from hundreds of millisecond to tens of second (Table S1). Compared to these studies, the duration of the produced shear flow from our single cavitation bubble is much shorter (~ 20 μs).

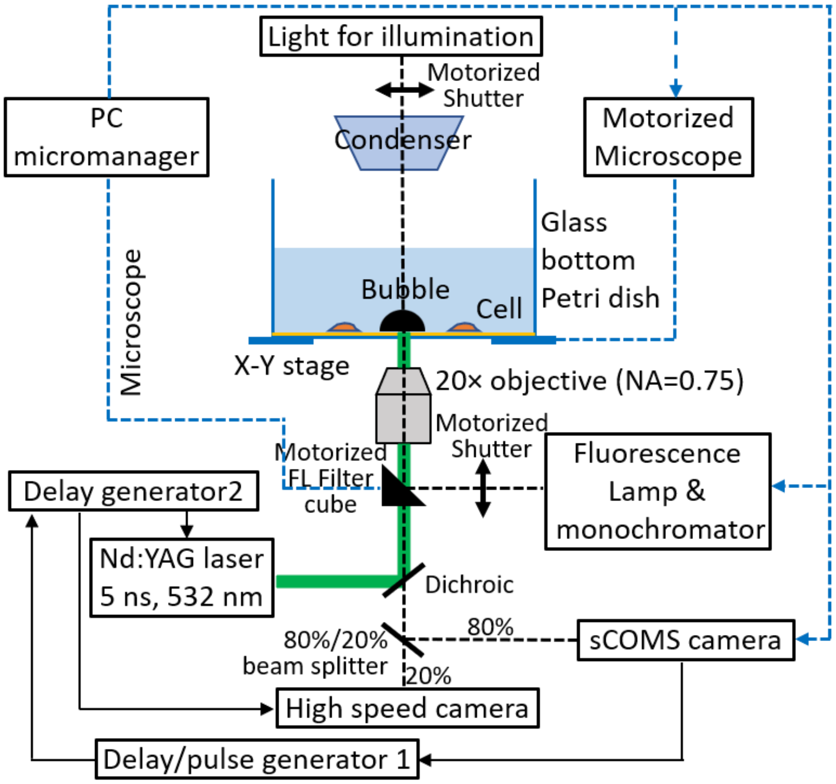

Figure S1. Experimental setup. Micromanager is utilized to synchronize the pulse laser (for SCB generation), motorized microscope system (Axio Observer Z1; Zeiss), fluorescence monochromator system (DeltaRAM X; PTI) and cameras (high speed camera and sCOMS camera) for imaging and data acquisition. The exposure out signal of the sCOMS camera (EDGE 5.5 CL; PCO) is used to trigger the delay / pulse generator 1, which controls the delay generator 2 at 10 s later to give command for the high-speed image acquisition and pulse laser firing. The fluorescence (FL) excitation, corresponding FL filter cube, and image recording were automatically switched between fura-2 and PI with a switching interval about 0.7s through μManager, which communicates and coordinates the operation sequence between the sCOMS camera, the microscope, the fluorescence monochromator, and the motorized shutters for bright field and fluorescence imaging, respectively. For more details, see Materials & Methods (Image recording and analysis) in the main text.

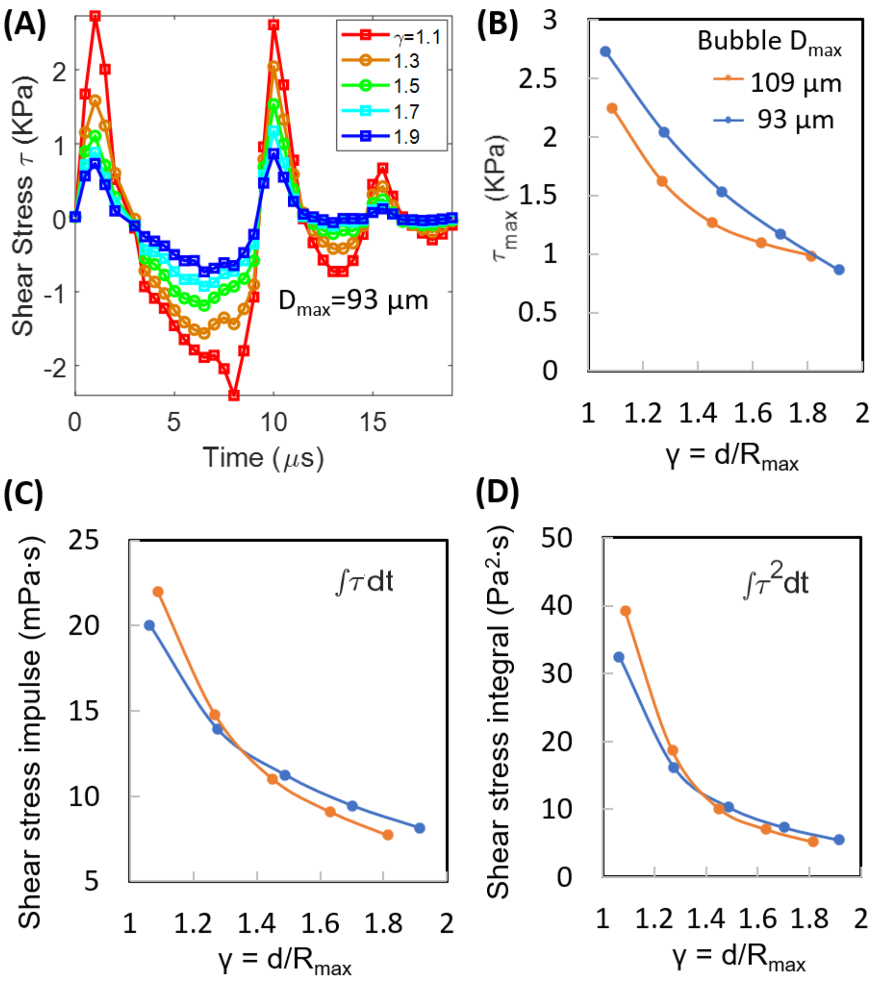

Figure S2. Estimation of SCB-generated shear stress near the substrate using the PIV data. (A) Time evolution of the shear stress τ at different normalized standoff distance γ using Eq.1 and PIV data. (B) The peak shear stress τ_{max}, (C) shear stress impulse ($\int \tau dt$) and (D) shear stress integral ($\int \tau^2 dt$) vs. γ for two bubbles representing the upper and lower limit of the bubble size used in our experiment.

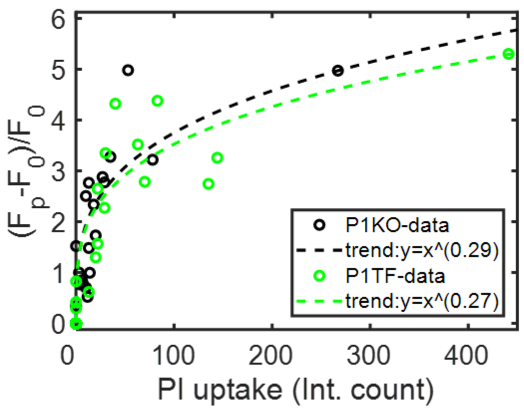

PIKO (γ=1-1.5)	Injury Ca^{2+} response	non-injury Ca^{2+} response	No Ca^{2+} response
**N**	**19**	**2**	**15**
**Percentage (%)**	**53**	**5**	**42**
**PITF (γ=1-1.5)**	**Injury Ca^{2+} response**	**non-injury Ca^{2+} response**	**No Ca^{2+} response**
**N**	**13**	**2**	**17**
**Percentage (%)**	**41**	**6**	**53**

Figure S3. Dependence of SCB elicited intracellular Ca^{2+} response on PI uptake and γ in P1KO and P1TF cells. *Left*: Correlation of Ca^{2+} response with PI uptake for the population of individual cells in the group of P1KO and P1TF among γ=1-1.5. Symbols represent data points measured from experiment while the dashed lines are power law trend of the data points, with R^2 of 0.9866 and 0.9949 for P1KO and P1TF, respectively. *Right*: a table showing the overall percentage for the three different kinds of cell response: Ca^{2+} response with injury, Ca^{2+} response without injury, no Ca^{2+} response and no injury in the range of γ=1-1.5.

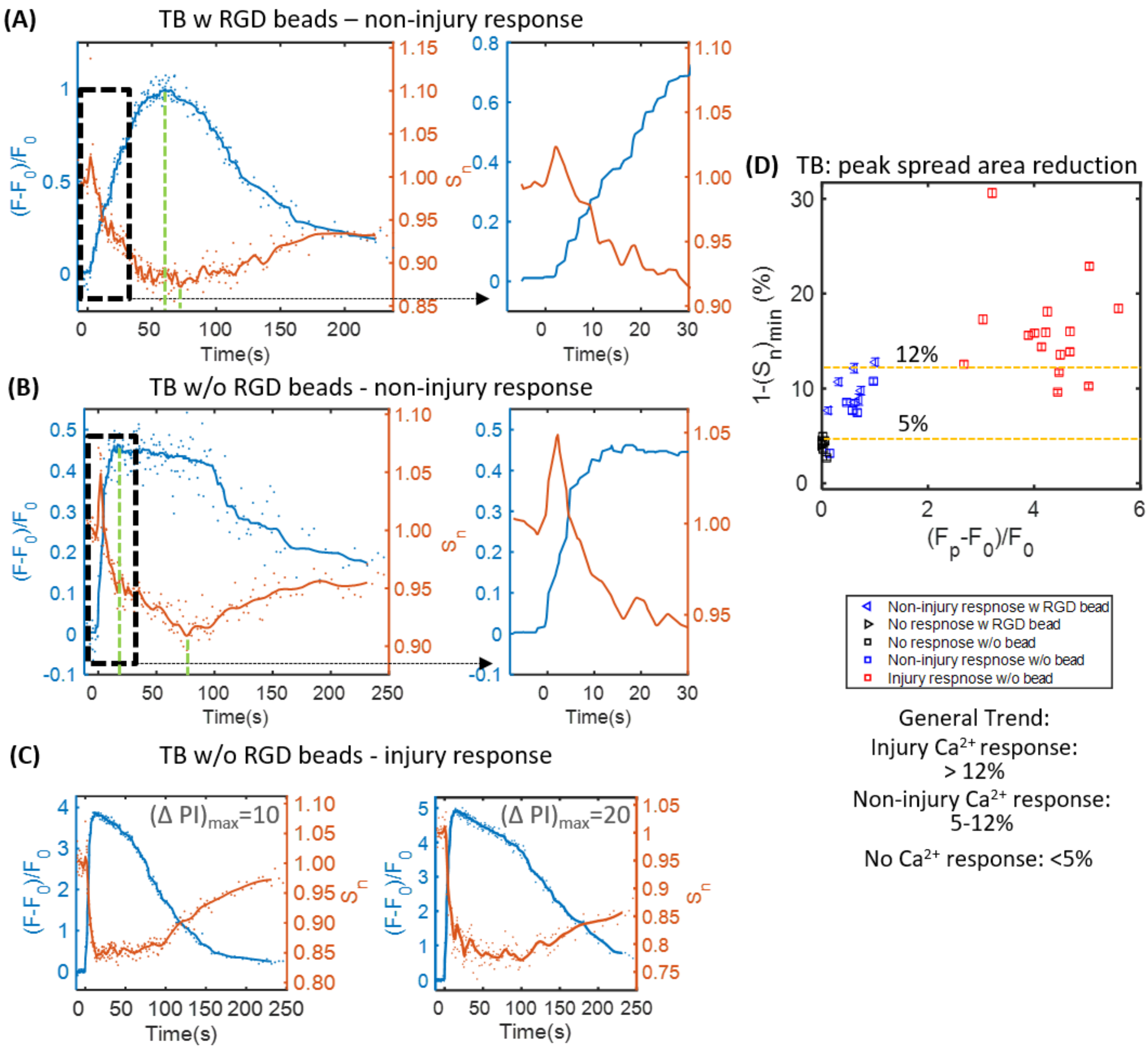

Figure S4. Transient increase and reduction of cell spread area for TB elicited different kinds of Ca^{2+} response in HeLa cells from our previous work [5]. (A) A representative HeLa cell with RGD beads treated by TB at S_d=60 μm. (B) A representative HeLa cell without RGD beads treated by TB at S_d=50 μm. *Left:* time traces of intracellular Ca^{2+} signaling (*blue*) and cell spread area alteration (*orange*). *Right*: enlarged view for the dashed box region on *left*, showing Ca^{2+} response is initiated during cell spread area increase immediately following TB treatment. (C) Time traces of intracellular Ca^{2+} signaling (*blue*) and cell spread area alteration (*orange*) for TB elicited injury Ca^{2+} response in two representative HeLa cells w/o beads and with different amount of PI uptake ($(\Delta PI)_{max}$), respectively. (D) Summary of the maximum cell spread cell reduction for TB evoked different kinds of Ca^{2+} response, either with RGD beads (*triangles*) or without beads (*squares*). The data shows that most cases of the injury Ca^{2+} response, non-injury Ca^{2+} response and no Ca^{2+} response has a maximum spread area reduction of above 12%, between 5% and 12%, and below 5%, respectively. The error bars depict the SEM.

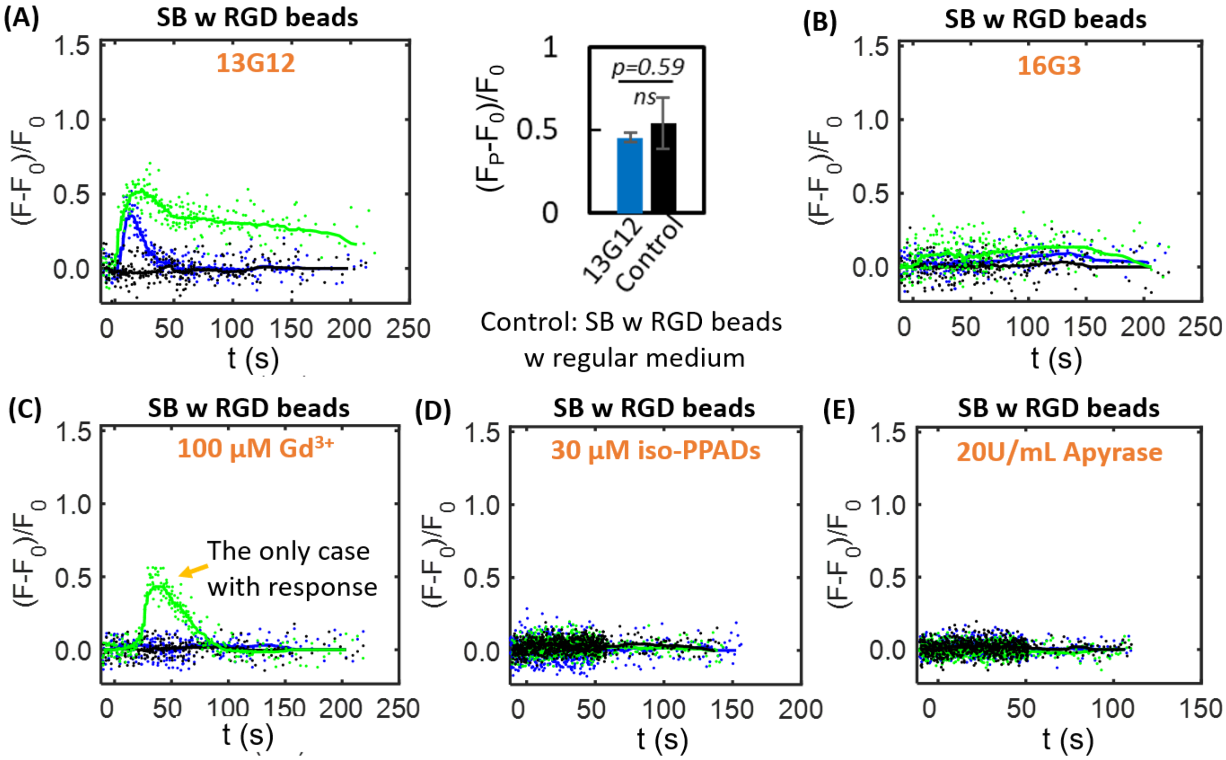

Figure S5. Representative time traces of Ca^{2+} response for the mechanistic study of RGD beads enhanced Ca^{2+} signaling. (A) and (B) HEK293T P1KO cells with RGD beads and treated with fibronectin antibody 13G12 (w/o blocking integrin ligation, 20 μg mL^{-1}) and 16G3 (blocking integrin-ligation, 20 μg mL^{-1}) when subjected to SCB induced flow, respectively. The bar graph in (A) shows the mean value of the amplitude of the normalized Ca^{2+} response $(F_p\text{-}F_0)/F_0$ with error bar indicating the SEM, demonstrating no significant difference between 13G12 treated group and the control group with regular cell medium (two-tailed t-test). (C) SCB treated P1KO cells attached with RGD beads with 100 μM Gd^{3+} in extracellular medium. The response case is the only cell with Ca^{2+} response among the whole treated population (N=15). (D) and (E) SCB treated P1KO cells that are attached with RGD beads and incubated with 30 μM iso-PPADs to block P2X channels and 20 U mL^{-1} apyrase to deplete extracellular ATP, respectively.

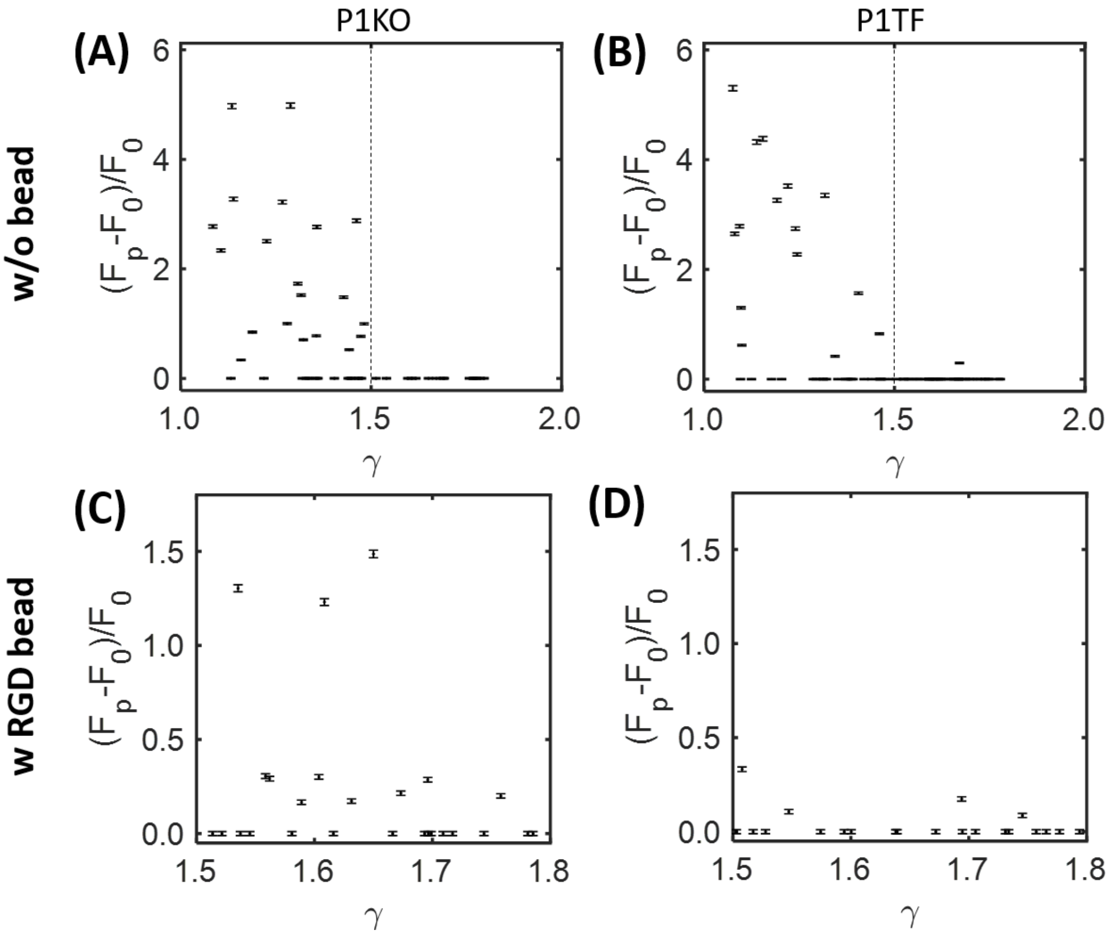

Figure S6. Error estimation for SCB induced Ca^{2+} response amplitude. (A) and (B) HEK293T P1KO and P1TF cells not treated with beads, corresponding to Figure 2F and Figure 2G in the main text, respectively. (C) and (D) HEK293T P1KO and P1TF cells treated with RGD coated beads, corresponding to the left and right panels in Figure 3E, respectively. The error bars show the SEM of the data.

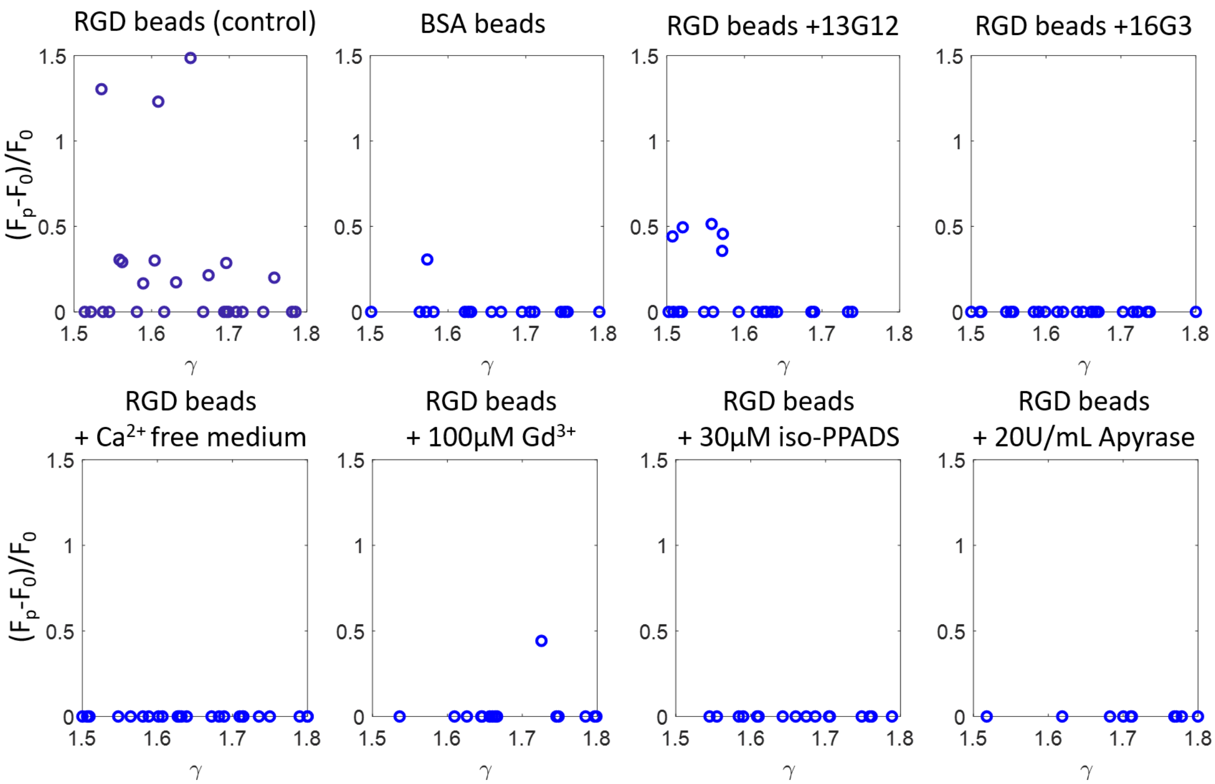

Figure S7. The dependency of cell Ca^{2+} response $(F_p-F_0)/F_0$ on the normalized standoff distance γ for the mechanistic studies shown in Fig. 6A&B. Overall, no strong dependence is observed within the non-injury range of γ = 1.5-1.8.

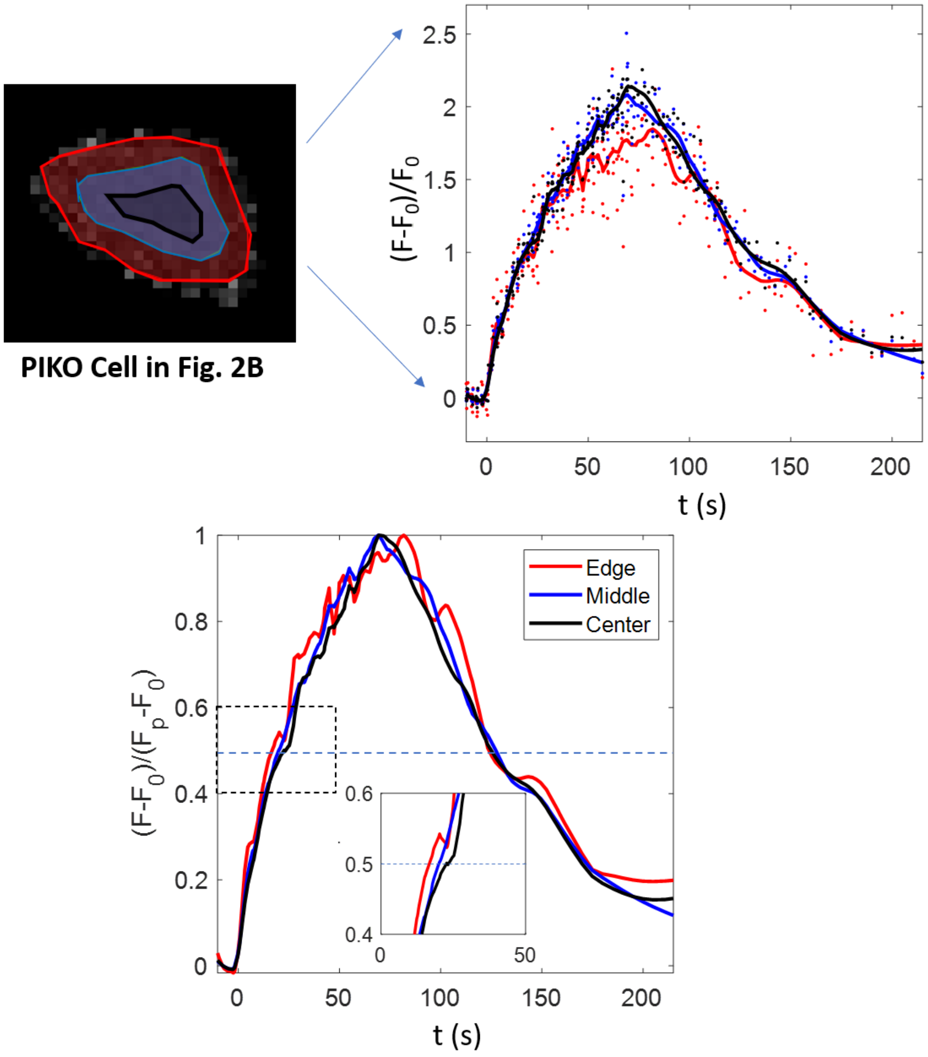

Figure S8. Small region analysis (edge, middle and center ROI) of the Ca^{2+} response for the cell in Fig.2B of the main text, which is not treated with microbeads. Top left: ROIs labels. Top right: Time traces of $(F-F_0)/F_0$ (dots) and median averaging fit lines. Bottom: A normalized version of the median averaging fit lines, an enlarged view is shown in inset for the dashed box around $(F-F_0)/(F_p-F_0)=0.5$ (horizontal dashed line).

Table S1. Summary on the activation of piezo1 from previous studies using ultrasound or shear flow.

Previous studies	Stimuli type	**f (MHz)**	Intensity or pressure	**Total treatment time**	PRF	duty cycle	Single pulse duration	Flow velocity and shear stress	cell system	Detection method for piezo1 activation	results
Prieto, M. L., et al. (2018) [8]	Continuous US	43	50 or 90 W/cm^2	**200 ms**	N.A.	N.A.	N.A.	Acoustic streaming velocity < 0.14 mm/s	HEK293T transfected with Piezo1	Electrophysiological Recording (patch-clamp)	Piezo1 is activated
Pan, Y., et al. (2018) [9]	US	2	~0.6 MPa	**5 s**	5Hz	10%	20ms	N.A.	HEK293T transfected w Piezo1	Ca^{2+} imaging	cannot activate Piezo1 unless with integrin-binding microbubble
Qiu, Z., et al. (2019) [10]	US	0.5	0.3 MPa	**200 ms**	1000 Hz	40%	400 μs	N.A	HEK293T transfected w Piezo1	Ca^{2+} imaging	Piezo1 can be activated
D.F. Liao, et al. (2019) [11]	VD-SAW	30	~1.6MPa	**60 s**	2Hz	20%	100ms	Streaming velocity of 0.8 m/s, shear stress 50 $dyne/cm^2$	HEK293T Piezo1 KO and transfected	Ca^{2+} imaging	Piezo1 can be activated
		30	~1.6MPa	**60 s**	200Hz	20%	1ms	Streaming velocity of 0.8 m/s, shear stress 50 $dyne/cm^2$	HEK293T Piezo1 KO and transfected	Ca^{2+} imaging	Piezo1 has no significant effect
Ranade, S. S., et al. (2014) [12]	Perfusion shear flow	Single pulse	N.A.	**600 ms**	N.A.	N.A.	600 ms	Average velocity of 6.9-21 mm/s, shear stress 23.3-70.2 $dyne/cm^2$	HEK293T transfected w Piezo1	Electrophysiological Recording (patch-clamp)	Piezo1 can be activated
This study	Laser-induced bubble	Single pulse	Bubble radius 45-55 μm	**~ 20 μs**	Single pulse	N.A.	~20 μs	Maximum velocity of 1.6-2.5 m/s (γ: 1.5-1.9); maximum shear stress 0.9-1.5 kPa (9000-15000 $dyne/cm^2$)	HEK293T Piezo1 KO and transfected	Ca^{2+} imaging	Piezo1 has no significant effect

SI References

1. Rau KR, Quinto-Su PA, Hellman AN, Venugopalan V. Pulsed laser microbeam-induced cell lysis: time-resolved imaging and analysis of hydrodynamic effects. Biophys J. 2006; 91: 317-29.

2. Yuan F, Yang C, Zhong P. Cell membrane deformation and bioeffects produced by tandem bubble-induced jetting flow. Proc Natl Acad Sci U S A. 2015; 112: E7039-47.

3. Grigioni M, Daniele C, Morbiducci U, D'Avenio G, Di Benedetto G, Barbaro V. The power-law mathematical model for blood damage prediction: analytical developments and physical inconsistencies. Artif Organs. 2004; 28: 467-75.

4. Arora D, Behr M, Pasquali M. A tensor-based measure for estimating blood damage. Artif Organs. 2004; 28: 1002-15.

5. Compton JL, Luo JC, Ma H, Botvinick E, Venugopalan V. High-throughput optical screening of cellular mechanotransduction. Nat Photonics. 2014; 8: 710-5.

6. Luo JC, Botvinick EL, Venugopalan V. Reply to 'Mechanism for microtsunami-induced intercellular mechanosignalling'. Nat Photonics. 2015; 9: 624-5.

7. Li F, Yang C, Yuan F, Liao D, Li T, Guilak F, et al. Dynamics and mechanisms of intracellular calcium waves elicited by tandem bubble-induced jetting flow. Proc Natl Acad Sci U S A. 2018; 115: E353-E62.

8. Prieto ML, Firouzi K, Khuri-Yakub BT, Maduke M. Activation of piezo1 but not NaV1.2 channels by ultrasound at 43 MHz. Ultrasound Med Biol. 2018; 44: 1217-32.

9. Pan Y, Yoon S, Sun J, Huang Z, Lee C, Allen M, et al. Mechanogenetics for the remote and noninvasive control of cancer immunotherapy. Proc Natl Acad Sci U S A. 2018; 115 (5): 992-997.

10. Qiu Z, Guo J, Kala S, Zhu J, Xian Q, Qiu W, et al. The mechanosensitive ion channel Piezo1 significantly mediates in vitro ultrasonic stimulation of neurons. iScience. 2019; 21: 448-57.

11. Liao D, Li F, Lu D, Zhong P. Activation of Piezo1 mechanosensitive ion channel in HEK293T cells by 30 MHz vertically deployed surface acoustic waves. Biochem Biophys Res Commun. 2019; 518: 541-7.

12. Ranade SS, Qiu Z, Woo SH, Hur SS, Murthy SE, Cahalan SM, et al. Piezo1, a mechanically activated ion channel, is required for vascular development in mice. Proc Natl Acad Sci U S A. 2014; 111: 10347-52.
